# Correction to: Towards Multi-Brain Decoding in Autism: A Self-Supervised Learning Approach

**DOI:** 10.1007/s12021-026-09805-1

**Published:** 2026-09-04

**Authors:** Ghazaleh Ranjbaran, Quentin Moreau, Adrien Dubois, Guillaume Dumas

**Affiliations:** 1 CHU Sainte-Justine Research Centre, Department of Psychiatry, Université de Montréal, Montréal, QC Canada; 2 Mila – Quebec AI Institute, Montréal, QC Canada

**Correction to: Neuroinformatics (2026) 24:6**

**https://doi.org/10.1007/s12021-025-09755-0**

The original online version of this article was revised. In this article the author name Ghazaleh Ranjbaran was incorrectly written as Ghazaleh Ranjabaran.

The original article has been corrected.

